# Genotyping, Orientalis-like *Yersinia pestis*, and Plague Pandemics

**DOI:** 10.3201/eid1009.030933

**Published:** 2004-09

**Authors:** Michel Drancourt, Véronique Roux, La Vu Dang, Lam Tran-Hung, Dominique Castex, Viviane Chenal-Francisque, Hiroyuki Ogata, Pierre-Edouard Fournier, Eric Crubézy, Didier Raoult

**Affiliations:** * Université de la Méditerranée, Marseille, France;; † Université de Bordeaux 1, Talence, France;; ‡ Institut Pasteur, Paris, France;; § Information Génomique et Structurale, Marseille, France;; ¶ Université Paul Sabatier, Toulouse, France

**Keywords:** Yersinia pestis, genotyping, historical plague, research

## Abstract

Three pandemics have been attributed to plague in the last 1,500 years. *Yersinia pestis* caused the third, and its DNA was found in human remains from the second. The Antiqua biovar of *Y. pestis* may have caused the first pandemic; the other two biovars, Medievalis and Orientalis, may have caused the second and third pandemics, respectively. To test this hypothesis, we designed an original genotyping system based on intergenic spacer sequencing called multiple spacer typing (MST). We found that MST differentiated every biovar in a collection of 36 *Y. pestis* isolates representative of the three biovars. When MST was applied to dental pulp collected from remains of eight persons who likely died in the first and second pandemics, this system identified original sequences that matched those of *Y. pestis* Orientalis. These data indicate that *Y. pestis* caused cases of Justinian plague. The two historical plague pandemics were likely caused by Orientalis-like strains.

## Abstract 2

Two historical plague pandemics were likely caused by Orientalis-like strains of *Yersinia pestis.*

*Yersinia pestis*, a group A bioterrorism agent (*1*), causes plague, a reemerging zoonotic disease transmitted to humans through flea bites and typically characterized by the appearance of a tender and swollen lymph node, the bubo (*2*). This organism has been subdivided into three biovars on the basis of their abilities to ferment glycerol and to reduce nitrate. Based on their current geographic niche and on historical records that indicate the geographic origin of the pandemics, researchers have postulated that each biovar caused a specific pandemic (*2**,**3*). Biovar Antiqua, from East Africa, may have descended from bacteria that caused the first pandemic, whereas Medievalis, from central Asia, may have descended from the bacteria that caused the second pandemic. Bacteria linked to the third pandemic are all of the Orientalis biovar (*3*). In this study, we tested this hypothesis for the first time by detecting biovars in ancient human remains.

No molecular biology–based method proved reliable and convenient for *Y. pestis* genotyping. Genome sequences of *Y. pestis* strain CO92, a Orientalis biovar, and *Y. pestis* strain KIM, a Medievalis biovar, are now available (*4**,**5*), which provides an opportunity to examine them for differences associated with the biovar and for genotyping. Genome analysis of the closely related *Rickettsia prowazekii* (*6*) and *R. conorii* (*7*) showed that intergenic spacers, which have been submitted to less evolutionary pressure than coding sequences, may be variable enough to differentiate closely related microorganisms. We, therefore, hypothesized that sequencing of several intergenic spacers would allow determination of a biovar-specific spacer pattern in *Y. pestis*. We named this method multiple spacer typing (MST). We first demonstrated that MST allowed biovar genotyping of a large collection of *Y. pestis* isolates and further applied it to the dental pulp collected from persons whose deaths are attributed to the first and second pandemics.

## Methods

### Bacterial Strains

Thirty-five strains representative of the three *Y. pestis* biovars (11 Antiqua isolates, 12 Medievalis isolates, and 12 Orientalis isolates) isolated from 1947 to 1996 from various host species in 13 countries are presented in Table 1. Nineteen of these isolates have been previously characterized by Achtman et al. (*8*). Nucleic acid was extracted as previously described (*9*), and species identification was confirmed for all the strains by partial sequencing of the *rpob* gene (*10*).

**Table 1:** Alleles of eight spacers in three *Yersinia pestis* biovars

Biovar	YP no. strains	Country	YP1	YP3	YP4	YP5	YP7	YP8	YP9	YP10	Isolate type
Antiqua											
	611/Japan	Japan	1	4	1	3	1	1	1	1	1
	552/Margaret	Kenya	1	3	1	1	4	1	1	1	2
	548/343	Belgium	1	3	1	1	5	1	1	1	3
	544	Congo	1	3	1	1	7	1	1	1	4
	549	Kenya	1	3	1	1	8	1	1	1	5
	542	Belgium	1	3	1	1	6	1	1	1	6
	550	Congo	1	3	1	1	9	1	1	1	7
	553	Kenya	1	3	1	1	7	1	1	1	4
	566	Kenya	1	3	1	1	6	1	1	1	6
	677	Kenya	1	3	1	1	9	1	1	1	7
	545	Kenya	1	3	1	1	7	1	1	1	4
Medievalis											
	519/PKH-4	Kurdistan	2	2	2	2	1	1	1	1	10
	616/PAR-13	Iran	2	2	2	2	1	1	1	1	8
	557/PKR292	Kurdistan	2	2	2	2	4	1	1	1	9
	564	Kurdistan	2	2	2	2	6	1	1	1	10
	565	Turkey	2	2	2	2	5	1	1	1	8
	557	Kurdistan	2	2	2	2	4	1	1	1	9
	518	Kurdistan	2	2	2	2	5	1	1	1	8
	520	Kurdistan	2	2	2	2	5	1	1	1	8
	560	Kurdistan	2	2	2	2	5	1	1	1	8
	561	Kurdistan	2	2	2	2	5	1	1	1	8
	617	Iran	2	2	2	2	5	1	1	1	8
	670	Kurdistan	2	2	2	2	5	1	1	1	8
	1594	Kurdistan	2	2	2	2	9	1	1	1	11
Orientalis											
	304/6-69	Madagascar	1	5	1	1	1	2	2	1	12
	685	Germany	1	5	1	1	2	2	2	1	13
	Hamburg10	USA	1	5	1	1	2	2	2	2	14
	CO92	USA	1	5	1	1	2	2	2	1	13
	507	Vietnam	1	1	1	1	6	2	2	1	15
	1513	Madagascar	1	5	1	1	2	2	2	1	16
	571	Brazil	1	5	1	1	4	2	2	1	17
	613	Myanmar	1	5	1	1	3	2	2	1	18
	643	Madagascar	1	5	1	1	3	2	2	1	18
	695	Germany	1	1	1	1	4	2	2	1	17
	772	Vietnam	1	5	1	1	4	2	2	1	17
	989	Vietnam	1	5	1	1	1	2	2	1	19

### Spacer Sequence Database and Phylogenetic Analyses

We analyzed the complete genome sequences of *Y. pestis* strain CO92, biovar Orientalis (GenBank accession no. NC-003143) (*4*) and *Y. pestis* strain KIM, biovar Medievalis (GenBank accession no. NC-004088) (*5*), which were obtained from the Kyoto Encyclopedia of Genes and Genomes (KEGG) database (*11*). We used the Primer3 program (http://frodo.wi.mit.edu/cgi-bin/primer3/primer3_www.cgi) to determine the primer sequences specific for the genomic segments of interest (*12*). The primers flanked intergenic sequences of *Y. pestis* CO92 that exhibited large sequence differences with the homologous *Y. pestis* KIM strain sequences. We generated a list of *Y. pestis* CO92 intergenic sequences of 50 to 300 bp and carried out BLASTN searches to identify the homologous intergenic sequences in *Y. pestis* KIM strain by using the *Y. pestis* CO92 genes flanking the intergenic sequences as queries (*13*). When both genes flanking the intergenic sequence exhibited best-matches with the BLAST score >120 bits, we estimated the length of the corresponding intergenic sequence in the *Y. pestis* KIM strain. We then aligned the homologous intergenic sequences (<300 bp) and selected eight pairs of sequences with insertion or deletion divergence between the two *Y. pestis* biovars to locate the primers (Table 2). Two microliters of DNA extracted as previously described (*9*) were amplified in a 50-µL mixture containing 10 pmol of each primer; 200 mmol/L (each) dATP, dCTP, dGTP, dTTP (Invitrogen, Cergy-Pontoise, France); 1.5 U *Taq* DNA polymerase (Invitrogen); and 2.5 µL of a 50-mM solution of MgCl_2_ in 1 x Taq buffer. Each polymerase chain reaction (PCR) was performed in a T3 thermocycler (Biométra, Archamps, France) under the following conditions: an initial 5 min of denaturation at 95°C was followed by 39 cycles of denaturation for 30 s at 94°C, annealing for 30 s at 60°C, and extension for 1 min at 72°C. The amplification was completed by holding the reaction mixture for 5 min at 72°C to allow complete extension of PCR products. These products were purified by using the Multiscreen PCR plate (Millipore Corp., Bedford, MA), as described by the manufacturer. Sequencing reactions were carried out with a DNA sequencing kit (Big Dye Terminator Cycle Sequencing V2.0; PE Biosystem, Courtaboeuf, France), as described by the manufacturer. Sequencing products were purified and underwent electrophoresis with the 3100 Genetic Analyzer (Applied Biosystems) and aligned by using the multisequence alignment CLUSTALX version 1.8 (*13*).

**Table 2:** Location of primers used for PCR amplification and sequencing of eight intergenic spacers in *Yersinia pestis^a^*

Spacer	Upstream gene (N) Downstream gene (N)	Primer sequence (5´ → 3´)
YP1	*ace* K (YP03724) *ace* A (YP03725)	AATCCCTGCAAAATGGTCTG CTGATGGGAAGCAAAGGTGT
YP3	*glg* P (YP03938) *glg* A (YP03939)	TCAGTGCATCCACACTGACA CGTATCGCCTTCACTAAGGC
YP4	gene ID 1176814 (YP03976) *gor* (YP03977)	TAATCCGCCGTGGAAATTAG ACGATTATCTGGCAATTGGC
YP5	Gene ID 1175557 (YP02727) Gene ID 1175558 (YP02728)	GCATGCGCTGTTTGATATTG TTATGACTCACGGACGATGC
YP7	*lex* A (YP00314) Gene ID 1173160 (YP00315)	GTAACGGGGACTGGATCTGA ATAAACCGTGTGCTTCCACC
YP8	Gene ID 1175559 (YP02729) Gene ID 1175560 (YP02730)	ACGGAAATTGCCAGATTCA GACTTGAGCTTCATTTGGCC
YP9	*mrd* F (YP02648) *mrd* E (YP02649)	GCGCTGATACGTGTTATTGG TTGTTAATATCGCGGGTGGTA
YP10	*bio D* (YP02269) Gene ID 1175101 (YP02270)	ATGCTGAAACAATCGCAATG CAATAAGGTGTACTCGCCGG

^a^Numbering according to the *Y. pestis* CO92 strain genome, GenBank accession no. NC-223143). PCR, polymerase chain reaction.

For phylogenetic analyses, DNA sequences were aligned by using the CLUSTALW software, version 1.81 (*13*). Because variations among spacer sequences were only due to deletions (see below), every deletion was considered a unique molecular event that resulted in a unique mismatch, regardless of its length. For any position in the spacer sequence, identity of nucleotide was coded "1"; a mismatch was coded "0"; and a pairwise binary matrix was constructed with the MEGA 2.1 software package (*14*). Distance matrices were determined by using p-distance analysis and were used to infer dendrograms by the unweighted pair group method with arithmetic mean (UPGMA), maximum parsimony, and neighbor-joining, available in the MEGA 2.1 software package (*14*). We also used the maximum likelihood method within the PHYLIP software package (*15*).

### Sources of Ancient DNA

**Figure A1:**
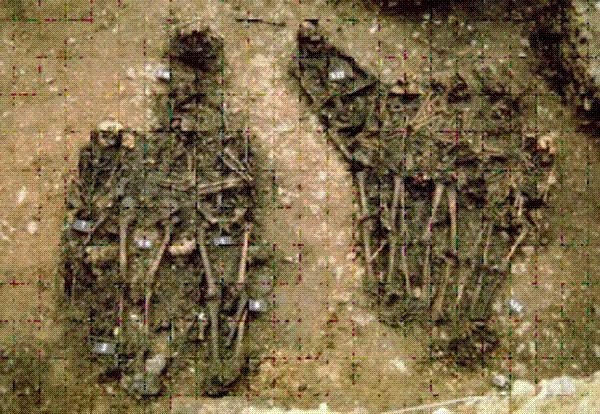
Partial view of the grave in Dreux investigated in this work, which illustrates anthropologic features of a mass grave suitable for paleomicrobiology research.

The Anthropology Laboratory of Bordeaux University 1 studied a first series of 60 skeletons discovered during excavations in 1989 in Sens, France. The skeletons were buried in four adjoining mass graves, dated by radiocarbon to be from the 5th to 6th century A.D (*16*). The second series came from a cemetery in Dreux, France, where nine mass graves were identified during excavations in 1990 (Figure A1). Each grave contained 2–22 skeletons. Ceramic fragments, found in the burial site, dated the graves from the 12th to the 14th century A.D (*17*). Archeological data (burial patterns) and anthropologic data (absence of bone fractures, indications of sex and age of persons) supported the hypothesis that the two grave sites contained remains of persons who died during an epidemic. No historical written records for the Sens and Dreux grave sites exist, but comparisons with demographic models suggest that the graves resulted from the Justinian plague (6th–8th century) and the Black Death, respectively. The Saint-Côme and Saint-Damien site in Montpellier had been used as a church cemetery outside the city walls during the 9th to 17th centuries A.D (*18*). Four graves have been dated as having been dug in the 13th and late 14th centuries, on the basis of their position on top of a 13th-century *remblai* (a small hill created by burying bodies) and the fact that they were behind a wall that dated from the second half of the 14th century. Dating of the different parts of this site was based on historical data, stratigraphy, the study of 7,059 ceramic fragments, and ^14^C dating.

We collected 10 teeth from three skeletons in Sens, 4 teeth from three skeletons in Dreux, and 5 teeth from two skeletons in Montpellier. The teeth were washed thoroughly with sterile phosphate-buffered saline and fractured longitudinally. Powdery remnants of dental pulp were scraped into sterile tubes for DNA extraction, as previously described (*19*). Seventeen teeth collected from contemporary dental patients in Marseille without any evidence of plague were used as negative control teeth ("negative teeth").

### Amplification of Ancient *Y. pestis*

**Figure A2:**
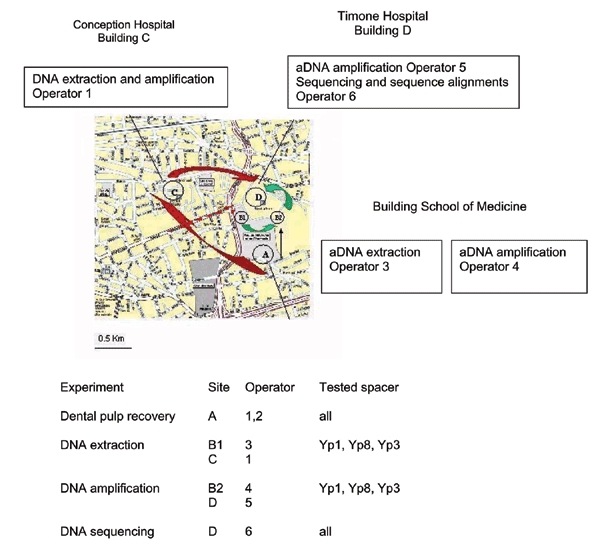
Detailed experimental process for ancient material to avoid *Yersinia pestis* contamination.

MST was applied to DNA extracted from 19 teeth from the remains of eight persons in one Justinian era burial and two graves from the time of the second pandemic, as described above. We instituted precautions to avoid bacteriologic and molecular contamination of ancient material (Figure A2). Two successive experiments were conducted. Dental pulp was recovered in a *Y. pestis*–free building A by two operators (operators 1 and 2), who had never worked with *Y. pestis*, its DNA, or amplicons. Teeth were processed individually by cleaning and opening the tooth and recovering the dental pulp into a sterile tube, which was closed securely. Each tooth was processed separately in chronologic order from Justinian to Black Death material, and negative control teeth were processed at the end. New sterile forceps were used for every tooth. Dental pulps were then transported into another laboratory, B1, 200 m away from building A, and to building C, 600 m away from building A for DNA extraction. It was performed by operator 3, who also had never worked with *Y. pestis*. DNA extraction for each experiment was performed with new reagents, ordered directly from the distributors by operators 1 and 3, who followed the protocol for good practices for ancient DNA manipulation (*20*). Extracted DNA was submitted to a second laboratory in building B2/D for suicide PCRs (*18*), which were conducted with one negative control for every three specimens. Finally, amplicons were transported to building D for cloning and sequencing, which was carried out by operators 4 to 6, who had never worked with *Y. pestis*. PCR products were cloned in PGEM-T Easy Vector (Promega, Charbonnières, France), as described by the manufacturer. Six clones were cultivated in LB medium (USB, Cleveland, OH) overnight, and plasmid purification was performed by using the Promega system. Six clones were sequenced from every amplicon by consensus primers. The sequences were compared with sequences available in GenBank by using the BLAST software version 2.2.8 to ensure accurate species identity. The sequences were further compared by using the BLAST software version 2.2.8 with our local *Y. pestis* spacer sequence database to ensure proper biovar identity.

## Results

### *Y. pestis* Spacer Database

All the isolates were firmly identified as belonging to *Y. pestis* species on the basis of their phenotypic profile and partial analysis of 16S rRNA gene and *rpo*B gene sequences. A total of eight intergenic spacers located throughout *Y. pestis* genome (Table 1) yielded 2–9 alleles per spacer, resulting in a total of 23 molecular events potentially corresponding to >8 x 10^6^ combinations (2^23^ molecular events). The 387-bp spacer YP1 exhibited a 183-bp deletion specific for the Medievalis isolates. For spacer YP3, we observed five alleles: Orientalis isolates 1513 and 695 exhibited a complete, 340-bp sequence, and the other Orientalis isolates exhibited a 16-bp deletion; Medievalis isolates featured a 48-bp deletion and a mutation G → A at position 135 of the spacer; Antiqua isolates exhibited a 32-bp deletion except for isolate 611, which had a 16-bp deletion and mutations C_164_ → T, C_166_ → T and A_191_ → G. Spacer YP3 thus differentiated every biovar. As for spacer YP4, Medievalis isolates were characterized by a 36-bp deletion, whereas Antiqua and Orientalis isolates exhibited a complete spacer. For spacer YP5, Medievalis isolates exhibited a full-length 292-bp spacer, whereas Antiqua and Orientalis isolates exhibited a 32-bp deletion at position 101 of the spacer. For spacer YP7, a total of nine alleles were found; Antiqua isolates were classified within seven alleles, Medievalis isolates within four alleles, and Orientalis isolates within five alleles. Alleles two and three were found only among Orientalis isolates. A full-length sequence of 330 bp was found for strain 549; the other isolates exhibited deletions. At position 126 in the spacer, strains 643 and 695 exhibited a 56-bp deletion; at position 133, strains 304, 1092, 507 and 571 exhibited a 49-bp deletion; at position 140, strains 611, 685, and 537 exhibited a 42-bp deletion; at position 142, strains 616, 548, 565, 518, 520, 560, 561, and 670 exhibited a 35-bp deletion; at position 149, strains 519, 542, 566, 564, and 1513 exhibited a 28-bp deletion; at position 155, strains 552, 613, 772, and 989 exhibited a 21-bp deletion; at position 162, strains 550, 677, and 1594 exhibited a 14-bp deletion; and at position 169 in the spacer, strains 544, 553, and 545 exhibited a 7-bp deletion. As for spacer YP8, Antiqua and Medievalis isolates exhibited a full-length 236-bp spacer, whereas Orientalis isolates had an 18-bp deletion at position 36 of the spacer. As for spacer YP9, Antiqua and Medievalis isolates exhibited a full-length 292-bp sequence; Orientalis isolates had an 18-bp deletion located at position 123 of the spacer. As for YP10 spacer, *Y. pestis* CO92 strain had a unique sequence of 369-bp; all the other isolates exhibited an 18-bp deletion located at position 177 of the spacer. Sequences herein determined were deposited in GenBank (Table A1).

### Phylogenetic Analysis

**Figure 1:**
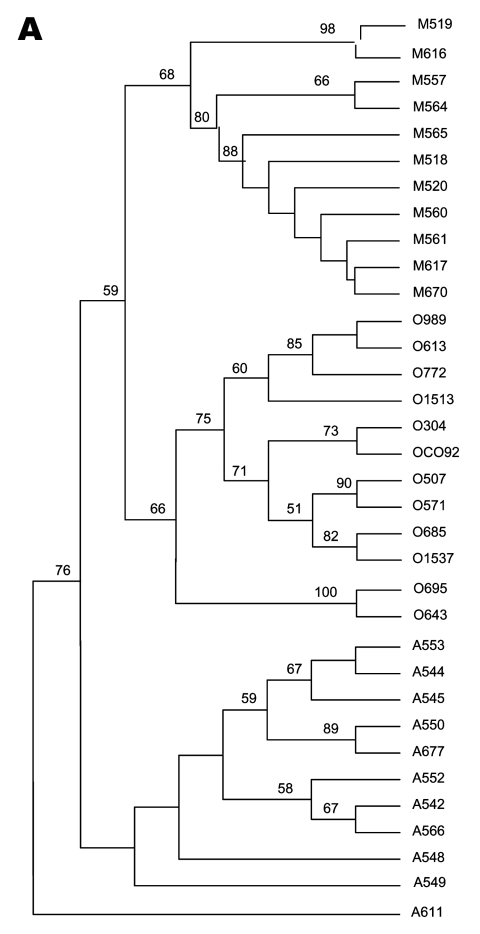
Unrooted tree showing the phylogenetic relationships among the 35 studied *Yersinia pestis* isolates inferred from sequence analysis of the combination of the eight intergenic spacers using the unweighted pair group method with arithmetic mean method. O, *Y. pestis* Orientalis biovar; M, *Y. pestis* Medievalis biovar; A, *Y. pestis* Antiqua biovar. Numbers refer to the isolate number as in Table 1.

**Figure A3:**
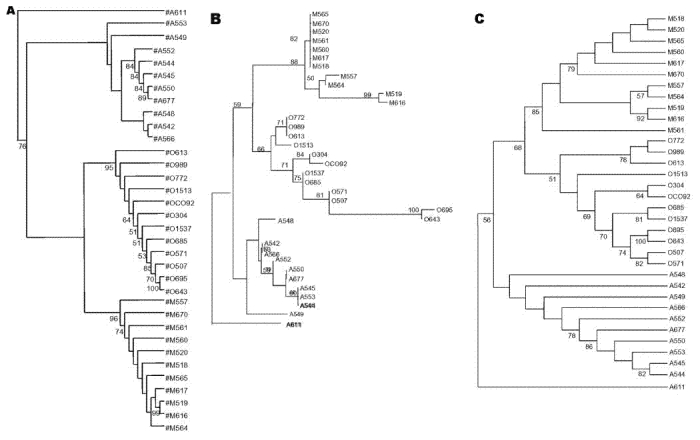
Unrooted trees showed the phylogenetic relationships among the 35 studied *Yersinia pestis* isolates inferred from sequence analysis of the combination of the eight intergenic spacers by using the parsimony (A), neighbor-joining (B), and maximum likelihood method (C).

Among the 35 studied *Y. pestis* strains, we identified three main phylogenetic clusters, each of which included only strains from a single biovar, i.e., Orientalis, Medievalis, or Antiqua, as observed in the unrooted dendrogram (Figure 1). The same topology was obtained when data were analyzed by parsimony, neighbor-joining, and maximum likelihood analyses (Figure A3). Within the Medievalis cluster, strains 519 and 616, and strains 557 and 564 were grouped by pairs; no other subgroup was identified. Within the Orientalis cluster, three groups were identified; one group included strains 989, 613, 772, and 1513; a second group was made up of three pairs of strains, i.e., 304 and CO92, 507 and 571, and 685 and 1537; the third group diverged before the differentiation of the other two groups and contained strains 695 and 643. Within the Antiqua cluster, two groups were identified; one group included strains 553, 544, 545, 550, and 677, with the last two clustered; the second group comprised strains 552, 542, and 566; strains 548 and 549 did not group with either of these two groups but diverged before the differentiation of the other Antiqua strains. The Antiqua strain 611 exhibited a unique phylogenetic position. This strain differed from all other studied *Y. pestis* strains and appeared to have diverged before the separation of the three main clusters.

### MST of Ancient Dental Pulp Specimens

**Figure 2:**
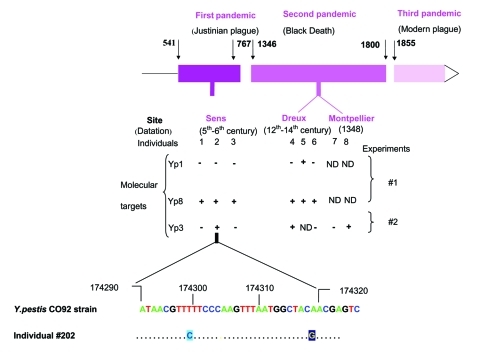
Molecular detection of *Yersinia pestis* was achieved in the dental pulp of remains of humans excavated from one Justinian and two Black Death mass graves in France by spacer amplification and sequencing (+, positive polymerase chain reaction [PCR] amplification and sequencing; –, absence of PCR amplification; ND, not done). Sequence analyses showed strains were of Orientalis genotype in all sets of remains; one of them exhibited two mutations numbered according to *Y. pestis* CO92 strain genome sequence (GenBank accession no. NC-003143). Negative control teeth remained negative.

In the 46 PCR experiments we performed on ancient tooth samples, we obtained 10 *Y. pestis* sequences (Figure 2); no sequences were found in the 51 PCR experiments with control teeth (p < 10^–4^). The teeth from 7 of the 8 persons' remains yielded 10 specific sequences, 3 persons were positive for two molecular targets, but none of the teeth of 17 persons used as negative controls yielded specific sequences (p < 10^–4^). YP1 PCR yielded an amplicon in one of six tested persons, YP8 PCR yielded an amplicon with identical sequence in six of six tested persons, and YP3 PCR yielded an amplicon in three of seven tested persons. When compared with GenBank database (Table A2), theYP1 sequence obtained in skeleton 5 yielded complete similarity with the homologous region in *Y. pestis* C092 strain over 390 positions and 99% sequence similarity with the homologous region in *Y. pestis* KIM strain over 174 positions; the YP8 sequence obtained in the six persons yielded 99% sequence similarity with homologous region in *Y. pestis* C092 strain over 178 positions and complete sequence identity with homologous region in *Y. pestis* KIM strain over 116 positions; the YP3 sequence obtained in skeletons 4 and 8 yielded complete sequence identity with that of homologous region in *Y. pestis* strain CO92 over 364 positions and 98% sequence similarity with homologous region in *Y. pestis* KIM strain over 206 positions; the YP3 sequence obtained in human remain 2 yielded a 98% similarity with homologous region in *Y. pestis* CO92 strain over 283 positions and a 95% similarity with homologous region in *Y. pestis* KIM strain over 162 positions. For each one of these 10 amplicons, further matches dropped to <90% sequence similarity over short sequences of 10 nt to 50 nt. When blasted to our local *Y. pestis* spacer sequence database, the YP1 spacer sequence obtained in human skeleton 5 was identical to that of the Orientalis and Antiqua reference sequences (Table A3), whereas smaller BLAST scores were obtained for the Medievalis reference sequences. Regarding the six YP8 spacer sequences obtained in skeletons 1–6, the 12 first best scores were obtained with Orientalis reference sequences. For the YP3 spacer sequence obtained in skeletons 4 and 8, the 10 first best scores were obtained with Orientalis reference sequences. For the YP3 spacer sequence obtained in skeleton 2, the 10 first best scores were obtained with Orientalis reference sequences, and this amplicon exhibited two specific nucleotide substitutions (Figure 2; Table A2). These mutations were consistently obtained in six clones.

## Discussion

Our data show that MST differentiates the three biovars of *Y. pestis* in a collection of 35 isolates representative of the three biovars and originating from various sources and 13 countries. This finding suggests that MST data can be extrapolated to the entire *Y. pestis* species. Pulsed-field gel electrophoresis (PFGE) has been the only other technique that allows for biovar and strain differentiation, but it requires large amounts of cultured microorganisms, and the stability of PFGE profiles in subculture has been questioned (*9*). Ribotyping did not classify isolates into their respective biovars (*9**,**21**,**22*). Specific insertion sequences (IS), including IS*100* (*23*), IS*285* (*24**,**25*), and IS*1541* (*26**,**27*), were used as markers in restriction fragment length polymorphism (RFLP) analyses (*8**,**28*) and in PCR-based technique (*29*). The last approach produced identical patterns of IS*100* distribution in Antiqua and Medievalis isolates (*29*). A variable-number tandem repeat technique (*30**,**31*) had a greater discrimination capacity than did ribotyping, but isolates from different areas were found to harbor identical types. Sequencing of fragments of five housekeeping genes in 36 *Y. pestis* isolates from various locations and from 12 to 13 isolates from *Y. pseudotuberculosis* and *Y. enterocolitica* did not show diversity in any *Y. pestis* housekeeping gene (*8*). Indeed, 19 of these 36 *Y. pestis* isolates were also included in the present work and featured 12 different MST profiles, thus demonstrating the validity of our hypothesis and the usefulness of MST for *Y. pestis* genotyping. Morever, its format is applicable to microbial analyses of ancient samples since it requires small amounts of DNA and targets small genomic fragments.

Few studies have aimed to disclose intraspecifc phylogenetic relationships of *Y. pestis* isolates. RFLP, probed with the IS*100*, indicated that, among 36 *Y. pestis* isolates, the three biovars formed distinct branches of the phylogenetic tree and that *Y. pestis* was a clone that evolved from *Y. pseudotuberculosis* 1,500–20,000 years ago (*8*). Isolates of biovars Antiqua and Medievalis clustered altogether apart from those belonging to biovar Orientalis. Likewise, a dendrogram constructed by the UPGMA clustering method on a PCR-based IS*100* fingerprint database clearly discriminated *Y. pestis* isolates of the Orientalis biovar that formed a homogeneous group, whereas isolates of the Antiqua and Medievalis biovars mixed together (*29*). Isolates of biovar Antiqua showed a variety of fingerprinting profiles, whereas Medievalis isolates clustered with the Antiqua isolates originating from Southeast Asia, which suggests their close phylogenetic relationships. In this study, one isolate (strain Nicholisk 51) displayed a genotyping pattern typical of biovar Orientalis isolates, although this isolate was biovar Antiqua and lacked a 93-bp deletion within the glycerol-3-phosphate dehydrogenase *glpD* gene characteristic for the glycerol-negative Orientalis biovar. Our data support the view that most *Y. pestis* isolates cluster according to their biovar, but isolates of biovar Antiqua are more distantly related to other isolates than biovars Orientalis and Medievalis are. MST-based phylogenetic reconstructions unexpectedly found that one *Y. pestis* Antiqua isolate, number 611, formed a fourth branch, which suggests that *Y. pestis* may comprise four different lineages instead of the three that have been recognized so far. This unique isolate had not been included in Achtman and collaborators' study (*8*).

MST was applied to ancient human specimens to test the hypothesis that three biovars were responsible for the three historical pandemics. Contamination of ancient samples by modern *Y. pestis* DNA and cross-contamination were prevented in our experiments. Indeed, we carried out two independent experiments, each with new reagents, in a laboratory where *Y. pestis* had never been introduced or studied, without positive controls (*17*).

**Figure A4:**
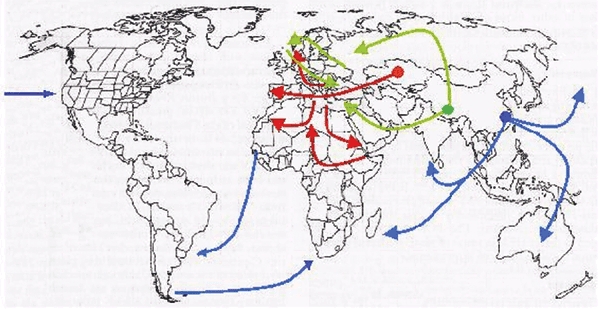
Geographic origin and routes of spreading of three historical plague pandemics labeled in red (Justinian plague), green (Black Death), and blue (modern), according to historical transcriptions reviewed in Perry (*2*).

Both experiments produced consistent results, and negative controls were always negative. That we obtained a unique YP3 sequence further excludes the possibility of contamination, since this sequence differs from all the currently known sequences. This unique sequence was consistently found in six of six clones and thus did not result from the false incorporation of nucleotides by the DNA polymerase. In our previous work on the Black Death, we also reported a unique sequence (*17*). In the present study, the accurate identification of *Y. pestis* was confirmed by using two successive sequence analyses. We first blasted the sequences derived from ancient specimens against the GenBank database and observed best matches for *Y. pestis* homologous sequences, thus ensuring accurate species identification of the amplicons. We then blasted these sequences against our local *Y. pestis* spacer sequence database and found best matches for homologous sequence in Orientalis reference isolates for the three tested spacers. As our local database has 35 different reference sequences representative of the three *Y. pestis* biovars, there is no doubt regarding the identification nor the fact that Orientalis-like *Y. pestis* alone was implicated in the personal remains that we investigated. DNA of *Y. pestis* was recovered from remains of persons in one mass grave established to be of the Justinian pandemic era on the basis of radiocarbon dating (Figure A4). We found that the genotype Orientalis, which now occurs worldwide, was involved in all three pandemics. Also, we detected *Y. pestis* in additonal human remains from Black Death sites, which adds more evidence for its role in the second pandemic in southern France (four sites tested positive) (*18**,**19*). Indeed, historical descriptions were suggestive of bubonic plague in medieval southern Europe; in northern Europe, historical data indicated that the Black Death had a different epidemiologic pattern. This finding may indicate that latter outbreaks in the north were not caused by transmission of *Y. pestis* by blocked rat fleas but rather by mechanical transmission of plague bacteria by another ectoparasite that used humans as their primary hosts. Alternatively, another pathogen may have caused these outbreaks, and a search for *Y. pestis* in the dental pulps of suspected plague victims in Copenhagen (two persons' remains) and Verdun (five persons' remains) dating from the 18th century failed to show *Y. pestis* DNA (*32*). Further studies may elucidate the respective role of *Y. pestis* and other pathogens that may have contributed to deaths in these times.

## Appendix

**Table A1:** Accession numbers

AY312282	*Yersinia pestis*	Antiqua	Strain #343	Yp1 spacer
AY312283	*Y. pestis*	Antiqua	Strain Margaret	Yp1 spacer
AY312286	*Y. pestis*	Antiqua	Strain Japan	Yp1 spacer
AY312284	*Y. pestis*	Medievalis	Strain #PKR 292	Yp1 spacer
AY312285	*Y. pestis*	Medievalis	Strain #PAR 13	Yp1 spacer
AY343088	*Y. pestis*	Medievalis	Strain PKH-4	Yp1 spacer
AY312287	*Y. pestis*	Orientalis	Strain Hambourg 10	Yp1 spacer
AY312288	*Y. pestis*	Orientalis	Strain C092	Yp1 spacer
AY312289	*Y. pestis*	Orientalis	Strain 6/69	Yp1 spacer
AY312292	*Y. pestis*	Antiqua	Strain #343	Yp3 spacer
AY312293	*Y. pestis*	Antiqua	Strain Margaret	Yp3 spacer
AY312295	*Y. pestis*	Antiqua	Strain Japan	Yp3 spacer
AY312294	*Y. pestis*	Medievalis	Strain PKR 292	Yp3 spacer
AY312291	*Y. pestis*	Medievalis	Strain PKH-4	Yp3 spacer
AY312296	*Y. pestis*	Medievalis	Strain PAR 13	Yp3 spacer
AY312290	*Y. pestis*	Orientalis	Strain 6/69	Yp3 spacer
AY312297	*Y. pestis*	Orientalis	Strain Hambourg 10	Yp3 spacer
AY312298	*Y. pestis*	Orientalis	Strain C092	Yp3 spacer
AY312328	*Y. pestis*	Antiqua	Strain #343	Yp8 spacer
AY312330	*Y. pestis*	Antiqua	Strain Margaret	Yp8 spacer
AY312331	*Y. pestis*	Antiqua	Strain Japan	Yp8 spacer
AY312326	*Y. pestis*	Orientalis	Strain 6/69	Yp8 spacer
AY312333	*Y. pestis*	Orientalis	Strain Hambourg 10	Yp8 spacer
AY312334	*Y. pestis*	Orientalis	Strain C092	Yp8 spacer
AY312327	*Y. pestis*	Medievalis	Strain PKH-4	Yp8 spacer
AY312329	*Y. pestis*	Medievalis	Strain PKR 292	Yp8 spacer
AY312332	*Y. pestis*	Medievalis	Strain PAR 13	Yp8 spacer
AY312354	*Y. pestis*	Orientalis	ancient specimen #202	Yp3 spacer
AY312357	*Y. pestis*	Orientalis	ancient specimen #283	Yp3 spacer
AY312356	*Y. pestis*	Orientalis	ancient specimen #292	Yp3 spacer

**Table A2:** Results of BLAST comparisons obtained with ancient *Yersinia pestis* sequences compared with GenBank^a^

GenBank accession no.	*Y. pestis* strains	Score (bits)	E value
AJ414158	CO92	773	0.0
AE013696	KIM	339	2^e-90^
AJ414156	CO92	339	2^e-90^
AE013666	KIM	337	9^e-90^
AJ414142	CO92	337	9^e-90^
AE013658	KIM	335	4^e-89^
AJ414143	CO92	335	4^e-89^
AE013654	KIM	333	2^e-90^
AJ414141	CO92	333	1^e-88^
AE013762	KIM	331	1^e-88^
AE014013	KIM	331	6^e-88^
AE013955	KIM	331	6^e-88^
AE013932	KIM	331	6^e-88^
AE013931	KIM	331	6^e-88^
AE013879	KIM	331	6^e-88^
AE013736	KIM	331	6^e-88^
AE013733	KIM	331	6^e-88^
AE013725	KIM	331	6^e-88^
AE013712	KIM	331	6^e-88^
AJ414160	CO92	331	6^e-88^

^a^Sequences in GenBank produced significant alignments with the Yp1 sequence from skeleton 5.

**Table A3:** Comparison of Yp1 spacer sequence derived from ancient specimens with our local spacer sequence database^a^

*Yersinia pestis* strains	Score (bits)	E value
Antiqua 545	773	0.0
Antiqua 677	773	0.0
Antiqua 566	773	0.0
Antiqua 553	773	0.0
Antiqua 544	773	0.0
Antiqua 550	773	0.0
Antiqua 542	773	0.0
Antiqua 549	773	0.0
Orientalis 1537	773	0.0
Orientalis 989	773	0.0
Orientalis 695	773	0.0
Orientalis 643	773	0.0
Orientalis 613	773	0.0
Orientalis 571	773	0.0
Orientalis 1513	773	0.0
Orientalis 507	773	0.0
Orientalis CO92	773	0.0
Orientalis 685	773	0.0
Antiqua 611	773	0.0
Antiqua 552	773	0.0
Antiqua 548	773	0.0
Antiqua 304	773	0.0
Medievalis 1594	305	4^e-86^
Medievalis 670	305	4^e-86^
Medievalis 617	305	4^e-86^
Medievalis 561	305	4^e-86^
Medievalis 560	305	4^e-86^
Medievalis 520	305	4^e-86^
Medievalis 518	305	4^e-86^
Medievalis 557	305	4^e-86^
Medievalis 565	305	4^e-86^
Medievalis 564	305	4^e-86^

^a^Sequences in our database that produced significant alignments are indicated.
